# Patients with COPD who underwent pulmonary rehabilitation in Turkey: prevalence, distribution, and mortality

**DOI:** 10.3906/sag-1901-224

**Published:** 2020-02-13

**Authors:** Tarkan ÖZDEMİR, İpek CANDEMİR, Pınar ERGÜN, Mustafa Hamidullah TÜRKKANI, Orhan KOÇ

**Affiliations:** 1 Department of Pulmonary Medicine, University of Health Sciences Dr. Abdurrahman Yurtaslan Oncology Education andResearch Hospital, Ankara Turkey; 2 Department of Pulmonary Medicine, University of Health Sciences Atatürk Chest Diseases and Chest Surgery Education and Research Hospital, Ankara Turkey; 3 Department of Pulmonary Medicine, Sincan State Hospital, Ankara Turkey; 4 Department of General Directorate for Disabled and Elderly Services, Ministry of Family and Social Policies, Ankara Turkey

## 1. Introduction

Chronic obstructive pulmonary disease (COPD) is defined as “a common, preventable, and treatable disease characterized by persistent respiratory symptoms and airflow limitation that are due to airway or alveolar abnormalities (or both), usually caused by significant exposure to noxious particles or gases” [1]. It is characterized by decreased exercise capacity, dyspnea, worsened quality of life, and exacerbations, which result in deterioration of these symptoms. As result of these factors, both the progression and economic burden of the disease becomes a serious problem. It has been shown to cause 2.9 million deaths per year worldwide, and is the third leading cause of global death [2]. 

Pulmonary rehabilitation (PR) is an effective, evidenced-based treatment modality in all symptomatic patients with chronic respiratory disease whose exercise capacity is reduced and quality of life is deteriorated, regardless of disease severity [1]. It has been demonstrated that PR is the most effective therapeutic approach for improving dyspnea, health status, and exercise tolerance [3]. Besides exercise capacity and quality of life, the evidence-based benefits are important for improving recovery time after hospitalization and reducing the perceived intensity of breathlessness, number of hospitalizations, and days in hospital. Additionally, it reduces hospitalizations among patients who have had recent exacerbations [1]. When considering these outcomes, it seems to be one of the most cost-effective therapeutic strategies. 

In Turkey, there are a limited number of pulmonary rehabilitation units although the number of patients with chronic respiratory problems is a growing burden. The prevalence of COPD in the population aged over 40 years was reported to range from 9.1% to 19.1% in Turkey [4–7]; a national disease burden report revealed that COPD was the third leading cause of mortality and the eighth leading cause of disability [8]. There are several international studies that show the rates of mortality in patients with COPD receiving PR, but there is a lack of data on mortality rates in those patients after PR programs in Turkey. There is only limited inconclusive evidence to show that PR has a significant beneficial effect on survival [9]. The aim of this study was to present the number of patients with COPD who underwent PR, general mortality percentages, the rate of patients prescribed PR by pulmonologists, and the distribution of institutions where PR was performed between 2008 and 2016 in Turkey. 

## 2. Materials and methods

### 2.1. Methods and data collection

This was a retrospective, observational, epidemiological, and descriptive study. The data of our study were obtained from the Turkish Institution of Social Insurance. Calculated data were obtained because of the Turkish Institution of Social Insurance’s security policy. The Turkish Institution of Social Insurance covers almost the entire population of Turkey (98.6%). The numbers of patients who were diagnosed with COPD and followed between 2008 and 2016 were scanned from the Oracle database using the TOAD data model program (version 9.6.0.27, Quest Software, USA). The total number of patients with COPD was recorded according to years. The age, sex, and number of patients with COPD who underwent PR between 2008 and 2016 were recorded. The study was approved by the local ethics committee.

### 2.2. Patients’ characteristics

The patients were diagnosed by a specialist. Patients aged 18 years and over were scanned, and the patients aged over 40 years were selected. The number of patients with COPD who underwent PR applications in different types of hospitals was identified. The general annual and general total mortality rates between 2008 and 2016 among patients with COPD who underwent PR in 2008 were also determined.

### 2.3. Definitions

General mortality was defined as death of unknown causes. Annual mortality was calculated by the proportion of patients who died within one year to the number of patients surviving at the beginning of the year (only calculated among the patients who underwent PR in 2008). Total mortality was defined as the proportion of the number of patients who died up to that year to the number of survivors in the beginning of 2008 (only calculated among the patients who underwent PR in 2008). 

### 2.4. Statistical analysis

SAS software (SAS Institute, Inc., Cary, NC, USA) was used for descriptive statistical analyses. Categorical variables were expressed as numbers and percentages (%).

## 3. Results

**Figure 1:**
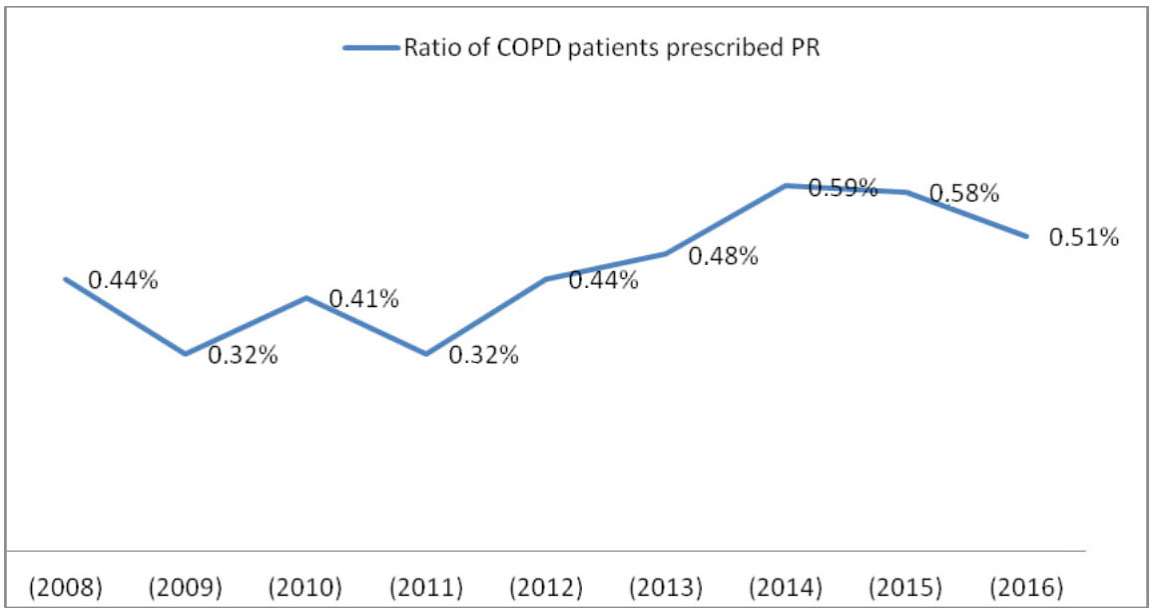
The ratio of patients with chronic obstructive pulmonary disease (COPD) who underwent pulmonary rehabilitation (PR) among all registered patients with COPD.

**Figure 2:**
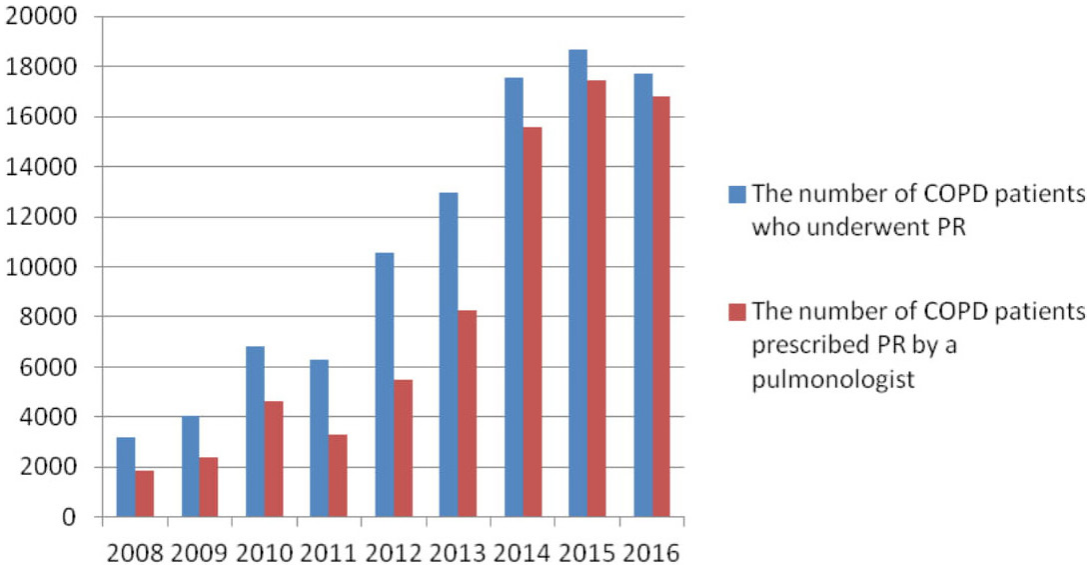
The ratio of patients with chronic obstructive pulmonary disease (COPD) who underwent pulmonary rehabilitation (PR) prescribed by a pulmonologist.

The number of patients diagnosed with COPD and followed in 2008 was 720,903, which increased to 3,434,262 in 2016. From 2008 to 2015, the number of patients with COPD who underwent PR increased progressively, from 3,214 to 18,664. In 2016, it was 17,707. The rate of patients who underwent PR was between 0.32% and 0.59% between 2008 and 2016. The lowest rate was 0.32% (n = 4,043 and 6,307 in 2009 and 2011), while the highest rate was 0.59% (n = 17,566 in 2014) (Table 1; Figure 1). Between 52.0% and 94.8% (5,488/10,549 and 16,792/17,707 patients, respectively) of the programs were prescribed by a pulmonologist between 2008 and 2016. The lowest rate was 52.0% (n = 5,488 in 2012), while the highest rate was 94.8% (n = 16,792 in 2016). Additionally, it appears that the rate rose progressively from 2012 to 2016 (Table 1; Figure 2). The number of male patients (n = 60,852, 62.1%) was higher than that of female patients (n = 37,018, 37.8%). The mean age ranges were 67.4 ± 12.3 to 72.0 ± 13.2 years (Table 1). The general annual mortality rates were between 6.2% and 11.1% (115/1,855 and 358/3,214 patients, respectively) in patients who underwent PR in 2008, and the general total mortality rate was found as 52.8% (1,696/3,214 patients) from 2008 to 2016 (Table 2). 

**Table 1:** 

Years	Total registeredpatients with COPD n	Patients with COPDwho underwent PR n (%)	Sex Male / Female n (%)	Age (years) Mean	Patients with COPD who prescribedPR by a pulmonologist n (%)
2008	720,903	3214 (0.44)	2,008 (62) / 1,206 (38)	72.0 ± 13.2	1,872 (58.2)
2009	1,228,182	4043 (0.32)	2,490 (62) / 1,553 (38)	70.4 ± 13.6	2,404 (59.4)
2010	1,638,303	6847 (0.41)	4,086 (60) / 2,761 (40)	69.6 ± 13.6	4,630 (67.6)
2011	1,962,833	6307 (0.32)	3,981 (63) / 2,326 (37)	69.6 ± 13.7	3,308 (52.4)
2012	2,344,777	10,549 (0.44)	6,322 (60) / 4,227 (40)	68.7 ± 14.1	5,488 (52.0)
2013	2,662,513	12,973 (0.48)	8,055 (62) / 4,918 (38)	68.7 ± 13.2	8,263 (63.8)
2014	2,946,215	17,566 (0.59)	10,801 (61) / 6,765 (39)	68.7 ± 12.9	15,598 (88.7)
2015	3,211,459	18,664 (0.58)	11,651 (62) / 7,013 (38)	67.8 ± 12.9	17,465 (92.5)
2016	3,434,262	17,707 (0.51)	11,458 (65) / 6,249 (35)	67.4 ± 12.3	16,792 (94.8)

**Table 2:** The general mortality rate of patients with chronic obstructive pulmonary disease (COPD) who underwent pulmonary rehabilitation (PR) in 2008.

	Patients	Annual mortality	Total mortality	n	n (%)	n (%)
	2008	3,214	358 (11.1)	358 (11.1)
2009	2,856	292 (10.2)	650 (20.2)
2010	2,564	217 (8.5)	867 (26.9)
2011	2,347	197 (8.4)	1,064 (33.1)
2012	2,150	148 (6.9)	1,212 (37.7)
2013	2,002	147 (7.3)	1,359 (42.3)
2014	1,855	115 (6.2)	1,474 (45.9)
2015	1,740	111 (6.4)	1,585 (49.3)
2016	1,629	111 (6.8)	1,696 (52.8)

**Figure 3:**
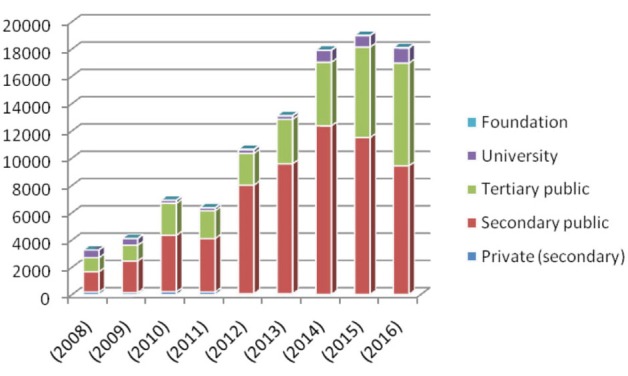
Distribution of patients with chronic obstructive pulmonary disease (COPD) who underwent pulmonary rehabilitation (PR) according to hospital type.

When the distribution of institutions where PR was performed was evaluated, the highest rates were in secondary public hospitals (n = 62,613, 62.9%), followed by tertiary public (n = 30,985, 31.1%), university (n = 4,838, 4.8%), private (n = 967, 0.9%), and foundation hospitals (n = 111, 0.1%), respectively. Although from 20011 to 2016 the number of patients with COPD who underwent PR applications in tertiary public hospital increased progressively, the rates were variable in university, secondary public, private, and foundation hospitals from 2008 to 2016. Furthermore, the number of patients who presented to private hospitals from 2008 to 2016 decreased (Table 3; Figure 3). 

**Table 3:** Distribution of patients with chronic obstructive pulmonary disease (COPD) who underwent pulmonary rehabilitation (PR) according to hospital type.

	2008n	2009n	2010n	2011n	2012n	2013n	2014n	2015n	2016n	Totaln (%)
Secondary public	1,481	2,318	4,146	3,912	7,957	9,526	12,357	11,494	9,422	62,613 (62.9)
Tertiary public	1,029	1,158	2,343	2,039	2,328	3,263	4,662	6,633	7,530	30,985 (31.1)
University	567	489	226	209	267	253	873	844	1,110	4,838 (4.8)
Private (secondary)	201	149	213	204	72	67	19	21	21	967 (0.9)
Foundation	0	4	3	4	29	18	13	10	30	111 (0.1)
Total number	3,278	4,118	6,931	6,368	10,653	13,127	17,924	19,002	18,113	99,514

## 4. Discussion

This is the first study to report the rates of patients with COPD who underwent PR in Turkey between 2008 and 2016, and to establish the number of patients with COPD who were prescribed PR by pulmonologists in the same period. This study showed that the rate of patients who underwent PR was about 0.32%–0.59%, there was a male predominance, and the mean age ranges were 67.4 ± 12.3 to 72.0 ± 13.2 years. The general annual mortality rates were 6.2%–11.1% in patients who underwent PR in 2008, and the general total mortality rate was 52.8% over the 9-year period in the same patient group. The number of patients with COPD who underwent PR was found to increase progressively. It was shown that PR was prescribed to more than half of the patients by pulmonologists. Additionally, PR was mostly performed in public hospitals.

Despite the proven benefits of PR, referral to PR programs still remains poor worldwide, regardless of the accessibility of PR [10–12]. Various rates have been reported; it is estimated that <5% of eligible patients received PR annually [13,14], but in some reports it has been found higher with rates of 3%–16% [15–19]. In their 2016 New Zealand audit, McNaughton et al. reported that only 2% of the expected COPD population was referred for PR, whereas in England and Wales, 68,000 (15.2%) of 446,000 eligible patients were referred [20]. A study investigated the frequency of referral for and attendance at PR among patients with COPD admitted to a tertiary Australian hospital in 2011; 57% of patients had been referred to PR at some stage of their disease, 18% had undergone PR at some point, and only 8% had received PR within the previous 2 years [21]. This referral rate was similar to that reported 10 years previously in a different Australian hospital, in a review of 49 patients with COPD [22]. Similarly, two studies from the United Kingdom reported referral to rehabilitation at the time of admission as 3% (42/1,400 patients) [23] and 18% (9/50 patients) [24]. It was emphasized that there was little change in referrals to PR in the last decade, in spite of the increased availability of programs and more widespread knowledge of effectiveness of PR. Keating et al. reported that uptake of PR had traditionally been poor, with up to half of the patients who were offered a course not attending PR sessions [25]. In our study, the rate of patients who underwent PR was about 0.32%–0.59% per year between 2008 and 2016. The lowest rate was 0.32% in 2009 and 2011, and the highest rate was 0.59% in 2014. Our rates were lower than in other studies. This could be due to the fact that our study consisted of a higher number of patients with COPD. We suggest that it was likely due to the low number of PR center and units and low awareness of PR among both health professionals and patients. In our study, there were more male patients than female in each year. The mean age ranges were 67.4 ± 12.3 to 72.0 ± 13.2 years. Similar to our study, in another study, the mean age of patients with COPD was 72 ± 11 years, and the majority was male, consistent with COPD features in the PR group [21]. 

In addition to the several benefits of PR described previously, PR has been associated with improved survival in patients with COPD after an acute exacerbation [26]; however, there are inconsistent data about improving survival in stable patients with COPD due to nonsignificant differences between mortality rates [27,28]. In a cohort study, survival was investigated in 158 patients who completed a PR program, 87% of whom had COPD, and the survival rate was 80% at 3 years after rehabilitation [29]. In other studies, survival rates were 91%–95% at 1 year [30,31] and 73% at 4 years after rehabilitation [30]. Another study demonstrated that the survival rate was 67% at 6 years in the PR group [28]. In our study, the general annual mortality rates were between 6.2% and 11.1% in patients who underwent PR in 2008, and the general total mortality rate was 52.8% over the 9-year period. 

Several factors affect attendance and referral to PR programs. Suboptimal healthcare professional awareness of PR is currently a barrier to patient referral [32]. In a recent study, it was shown that one of the most important reasons for nonattendance or referral was a lack of knowledge of the effectiveness of PR [4]. For the purpose of increasing referrals of patients to PR, the awareness of PR among healthcare professionals should be increased, along with that of patients. Education and training programs are recommended even for pulmonologists in recent guidelines [33]. In a study conducted in Turkey, primary healthcare providers such as family practitioners and homecare staff were found to have inadequate information about COPD and PR [34]. In a Turkish survey study, it was reported that the levels of knowledge of chest physicians about PR was substantially in the range of low to moderate in a small city of Turkey [35]. In our study, the rates of patients with COPD whose programs were prescribed by pulmonologists were 50% and over from 2008 to 2016. It seems that the awareness of pulmonologists increased progressively from 2012 to 2016. In 2016, the rate was 94.8%.

Another possible factor that influences attendance and referral to PR programs is health systems and policies. In our study, when the distribution of institutions where PR was performed was evaluated, the highest rates per year were in secondary public hospitals. Secondary public hospitals were followed by tertiary public, private, and university and foundation hospitals, respectively. Although the number of patients with COPD who underwent PR applications in tertiary public hospitals from 2011 to 2016 increased progressively, the rates were variable in university, secondary public, private, and foundation hospitals from 2008 to 2016. Furthermore, the number of patients who presented to private hospitals from 2008 to 2016 decreased. Since a patient may present to more than one health institution, the calculated number of patients with COPD who underwent PR would be higher in terms of institutional distribution.

The limitation of this study is the lack of information about the duration and content of PR programs and the number of PR centers/units. Due to the lack of a definition of PR centers and units in Turkey, these data could not be obtained from the Turkish Institution of Social Insurance. The data for every patient could not be obtained because of the information security policy of the Turkish Institution of Social Insurance. The records in the Turkish Institution of Social Insurance began in 2017. Therefore, the number of patients diagnosed with COPD in 2008 may be lower than expected. 

In conclusion, this study showed that although the awareness of PR, especially in pulmonologists, and the number of patients with COPD who underwent PR tended to increase in public hospitals, PR was still an underutilized approach in Turkey between 2008 and 2016. The awareness of PR should be increased in our country. In order to achieve this, we think that PR should be within the scope of health policies. Further studies are needed to identify referral problems and reasons for the lack of awareness of the effectiveness of PR. 

## Informed consent

Manuscript reporting does not include experimental investigations conducted with humans.
